# Experience of Managing Countertransference Through Self-Guided Imagery in Meditation Among Healthcare Professionals

**DOI:** 10.3389/fpsyt.2022.793784

**Published:** 2022-02-17

**Authors:** Olaug Julie Aasan, Hildfrid Vikkelsmo Brataas, Bente Nordtug

**Affiliations:** ^1^Division of Mental Health and Addiction, Oslo University Hospital, Oslo, Norway; ^2^Faculty of Nursing and Health Sciences, Nord University, Levanger, Norway

**Keywords:** therapeutic relationship, countertransference, meditation, positive imagery, wellbeing, self-care, imagery rescripting, healthcare professionals

## Abstract

**Introduction:**

As a part of the therapeutic relationship, a significant, well-established predictor of outcomes in psychiatric healthcare, healthcare professionals' emotional reactions to patients may affect treatment outcomes.

**Aim:**

The aim of our study was to explore and describe healthcare professionals' experiences with managing countertransference using skills from a training program on self-guided imagery in meditation (SIM).

**Method:**

Following an exploratory descriptive design, we conducted qualitative interviews with 10 healthcare professionals who care for patients with mental illness and subjected the collected data to thematic content analysis.

**Results:**

Participants reported that SIM had helped them to manage countertransference and had prompted changes that we categorized into three themes: managing personal vulnerability, setting clearer boundaries, and practicing self-care.

**Conclusion:**

The results suggest that by cultivating wellbeing and dealing with unresolved inner conflicts, SIM can help healthcare professionals to manage countertransference.

## Introduction

An established predictor of outcomes in psychiatric healthcare, the therapeutic relationship involves applying the concepts of helping relationships as well as of therapeutic, working, and helping alliances. Because the therapeutic relationship is integral to support and treatment services targeting people with mental illness (1–4), the concept of the relationship is a core component of professional psychiatric healthcare (5). In fact, studies have even suggested that clinicians' emotional reactions to patients can affect the outcomes of treatment (6–9).

Relationship competency is a prerequisite for establishing a therapeutic relationship (10). Rodolfa, Bent (11) have defined relationship competency as a foundational capacity “to relate effectively and meaningfully with individuals, groups, and/or communities” (p. 351). Beyond that, Gelso and colleagues have delineated the configuration of patient transference and therapist countertransference as one of three elements of the therapeutic relationship (12–14).

Health care professionals' reactions of countertransference have received sustained attention as an important aspect of the therapeutic relationship (15–18). First presented by Sigmund Freud, the term and classical definition of countertransference referred to the therapist's unconscious reactions to a patient's transference (19). In that narrow view, the therapist's countertransference is considered to be a hindrance to treatment. Since then, the concept of countertransference has been defined in various ways (7), although three conceptions seem most prominent: the aforementioned classical view as well as the totalistic view and the complementary view (20). The totalistic view includes all of the emotional reactions, both conscious and unconscious, that therapists have toward their patients (16, 21). In that view, countertransference is inevitable and partly related to the patient's inner world. By contrast, the complementary view, albeit somewhat overlapping the totalistic view, conceives countertransference as a counterpart to the patient's style of relating (15, 22). Taken together, the three views underscore that clinicians and theorists have recognized the importance of countertransference reactions in developing and maintaining appropriate therapeutic relationships between therapists and their patients. All three views also emphasize the potential influence of therapists' emotions and unresolved neurotic conflicts, including childhood experiences, when working with patients (7, 23). Hayes et al. (24) have even suggested that countertransference seems ubiquitous and should be regarded as a pan-theoretical construct.

Because a clear definition of countertransference has yet to be developed (18), we borrowed heavily from the totalistic view and defined countertransference for our study as all of the emotional responses and reactions that a healthcare professional may have toward a patient, shaped by the professional's learned beliefs, lived experience, and schemas as well as the transference materials presented by the patient. That definition incorporates the professional's reactions, both conscious and unconscious, to both transference and non-transference materials presented by the patient, reactions that may manifest as affective, cognitive, somatic, and/or behavioral responses. In line with Gelso and Hayes (25), we view countertransference as being inevitable, for all therapists have unresolved unconscious conflicts and “soft spots” or vulnerabilities that are touched upon in interactions with other human beings. Those authors also point out that triggers for countertransference are usually external to the therapist and can include the patient's personality style, the content presented by the patient, and even the patient's physical appearance (26).

The view that patients have common characteristics that universally provoke countertransference has not been supported by all research (27–31). From that standpoint, countertransference is viewed as subjective and dependent upon therapists' individual histories, conflicts, and vulnerabilities rooted in their life experiences, as well as learned beliefs that influence their interpretation of current interactions. In either case, newer studies have indicated that countertransference reactions may contain valid clinical information. For example, clinicians working with patients with narcissistic personality disorder reported feeling devalued and criticized by the patients; experiencing anger, resentment, and dread; and finding themselves distracted, avoidant, and wanting to terminate the treatment (32). Research has additionally revealed statistically significant variance in therapists' emotional reactions attributable to patients' specific personality pathologies (32–35). On the other side, patients are often aware of, and may directly experience, manifestations of countertransference or other behaviors, such as unresponsiveness, sarcasm, and confrontation (36).

According to Hayes et al. (24), meta-analytic evidence suggests that acting out due to countertransference is typically harmful, though not necessarily irreparable. For example, a healthcare professional may manifest disengagement by consistently making brief appointments, always standing by the door, or frequently checking their wristwatch when talking with a particular patient. Based on their rather general conclusions, Hayes et al. (24) recommend several clinical practices to help therapists to manage countertransference and prevent enactments. It seems particularly important for therapists to continually cultivate self-insight, seek to know themselves via impartial, honest, persistent self-observation and self-awareness, and to foster an understanding of their own and others' blind spots. They also advise therapists to tend to their psychological health, including by enforcing healthy boundaries with patients, and to practice self-care (24)—for instance, by getting enough sleep, limiting the number of patients whom they see, spending time with friends, maintaining a healthy diet, exercising regularly, and focusing on the rewards of counseling therapy—all of which are behaviors associated with resilience and ultimately better psychotherapy outcomes (24, 37). Added to that, Geller et al. (38) have emphasized the importance of having therapists resolve major conflicts in their own lives, which highlights the potential value of personal therapy for therapists.

Therapists are likely to benefit from engaging in regular, sustained meditation (38). Walsh and Shapiro (39) have defined meditation as “a family of self-regulation practices that focus on training attention and awareness in order to bring mental processes under grater voluntary control, and thereby foster general mental wellbeing and development and/or specific capacities such as calm, clarity, and concentration” p. 228–9. By extension, Davis and Hayes (40) have explained that meditation promotes healthy psychological and neurophysiological benefits, including emotion regulation, while qualitative and quantitative research has shown that meditation benefits the management of countertransference (41, 42). One way to manage countertransference may be using imagery techniques that involve not only positive imagery, which entails creating goals, developing new skills, and problem-solving, but also imagery rescripting (43).

Imagery rescripting is the technique of addressing specific memories of past experiences associated with present problems. In the technique, the person is instructed to recall an aversive experience and to rescript an imagined change in the memory or fantasy of the experience (43, 44). To that end, the therapist should led the person to first remember the aversive experience as vividly as possible, as if it were happening in the moment, and next imagine a new sequence of memory or fantasy for the experience in the direction that the person desires (44, 45). In that way, a childhood experience of being bullied in school or being rejected by peers, for example, can be modified via imagery; perhaps an older peer with higher social status enters the situation and invites the person to play, thereby introducing the new emotional experience of being safe and valued by a significant other.

By contrast, studies have shown that self-guided imagery in meditation (SIM), performed without a therapist's guidance, improve emotional self-regulation, perceived self-efficacy, satisfaction with life, and meaningfulness in life as well as reduce depressive tendencies (46, 47). Those same studies demonstrated the effect of learning SIM by participating in a 2-day, lecture-based program on structures of consciousness and guided meditation and applying the knowledge gained for 12 weeks thereafter. In the program, participants were taught to use SIM for four primary reasons, the first being to turn a past psychotraumatic event into a positive one via imagery rescripting, thereby helping them to cope with past psychotraumatic events or to change inexpedient learned beliefs. To that purpose, prior to imagery rescripting, participants were instructed to enter a meditative state and, in turn, search their minds for an idea of what has caused their current problem. The second reason was to achieve goals by creating positive imagery of future events, whereas the third was to improve social interactions by also creating positive imagery of them and changing emotions meeting with others. The fourth and final reason was to enhance one's emotional balance in daily life by learning to visualize the next day in a positive light.

Because meditation can increase access to information in the unconscious (48, 49), using SIM might also afford access to such information. Although practicing SIM may affect relational competence (46, 47), it remains unknown whether psychiatric healthcare professionals relate to patients more effectively and meaningfully when employing SIM. Thus, in a sample of healthcare professionals providing care for patients with mental illnesses, we investigated whether SIM has been experienced as a potential source of support with managing countertransference. To that end, our research question was: Have healthcare professionals providing care for patients with mental illnesses experienced SIM as helping their management of countertransference and, if so, then in what ways has SIM helped?

## Methods

Following an exploratory descriptive design in our qualitative study, we aimed to explore and describe experiences healthcare professionals' use of SIM to help their management of countertransference. Interviews with 10 healthcare professionals who use SIM were subjected to content analysis following an approach inspired by what Vaismoradi et al. (50) and Graneheim and Lundman (51) have described. Although both manifest content analysis and latent content analysis involve interpretation, such interpretation varies between them both in depth and in the level of abstraction. Whereas, manifest content analysis is concerned with visible components on the surface of the text (i.e., what the text says), latent content analysis is concerned with the meaning beneath the surface (51, 52).

### Sample and Sampling

We recruited healthcare professionals from different regions of Norway who had attended a 2-day training program on SIM. For inclusion criteria, all participants had to work in positions that involve providing care to people with mental health illness and had to have attended the 2-day program on SIM in the past 5 years and thereafter used the SIM techniques learned. Prospective participants received information about the study in a letter sent via a participant registry for people who had attended the training program. The first 10 professionals who volunteered to participate and who met the inclusion criteria were interviewed.

The participants included two mental health nurses, one registered nurse, two doctors, two nursing assistants, two social workers, and one professional health coach. By age, they ranged from 23 to 58 years old, and all were women. Their duration of work experience in the field varied from 1 to more than 20 years, for a mean length of 11 years, and all either in specialized or community healthcare services. Although some had completed the training program several times, others had attended only once, and none had received additional training in psychotherapy. The frequency with which the participants had used SIM after attending the program varied from rarely to more than once daily. On the whole, recruiting participants with various experiences increased the possibility of answering the research question from various participants' perspectives (51), and in that regard, the sample illuminated varied use of SIM.

### Individual Interviews

Seven semi-structured in-depth interviews were conducted in the participants' homes at their request, whereas the other three participants opted to be interviewed via video call. The interview guide was based on knowledge in the field and field-tested in a pilot interview (53).

At the beginning of the interviews, all participants were asked to describe how they have used SIM techniques to check for their compliance with the SIM method. The interview guide further invited participants to reflect on their use of SIM in relation to their relationship with patients, their communication with them, and their emotional reactions to them. The duration of the interviews ranged from 25 min to 2.5 h. The interviews were audiotaped and transcribed verbatim by the first author. In quotations in this manuscript, “...” indicates a brief pause.

### Analysis

Using content analysis, we aimed to examine whether participants had experienced that using SIM had helped them to manage countertransference and, if so, then how, as well as to describe that phenomenon in a conceptual form (50). After all interviews were conducted and transcribed, the transcripts were analyzed one at a time. Because no previous studies have addressed experiences with using SIM in relation to countertransference, we performed inductive content analysis, in which the coded categories are derived directly from the text (50).

In the first step of analysis, the text was read several times to obtain an overall impression. Second, meaning units relevant to the aim of the study were identified and abstracted into condensed meaning units. We proceeded with manifest content analysis. Third, the condensed meaning units were coded, as exemplified in Table 1.

**Table 1:** Example of a meaning unit, condensed meaning unit, and code.

**Meaning unit**	**Condensed meaning unit**	**Code**
“I'd be so tired when I left the office that I would fall asleep.... There's a group who drained me a lot, and at the same time, I had so many other feelings as well. I had a need to help them … they never get help. They stay, and I can never help them sort of… It's a very hard group, and it's a group that's triggered me a lot. Then [after practicing SIM]... suddenly I realized that I'm relating to them differently.”	The participant felt drained by a certain group of patients, was often triggered, and experienced that she related to the group differently.	Managing fatigue

After that, the various codes were compared for similarities and differences and sorted into tentative subcategories, which were subsequently analyzed and developed into categories. A category answered *what* was experienced and could be identified as a thread throughout the codes (51). To orient the analysis of subsequent transcripts, the table of categories from the former transcripts was used.

Analysis focused on whether the meaning of the statements matched with or further articulated the categories in the table or else addressed a new category. No new information emerged when analyzing the tenth interview, which we considered to indicate data saturation, meaning that no new information could be expected to surface from further analysis (54).

The first author carried out the first phase of analysis while discussing the analysis with the third author. Next, the latent content of the categories was formulated into three descriptive themes by all three researchers. In line with Graneheim and Lundman (51), a content analyst may use manifest content in developing categories and latent content when developing themes.

Last, all three researchers collaborated on a critical review of the analysis. Achieving consensus among co-researchers is one way to demonstrate credibility (52), which we did in an effort “to show the logic in how categories and themes are abstracted, interpreted, and connected to the aim and to each other” (p. 29). All three researchers also discussed the research question, applicable theory, the literature, and the final writing of a structural description. To further enhance credibility, we have presented representative quotations from the data with our results.

### Ethics Statement

Participants were assured full anonymity and were free to withdraw from the study at any time for any reason without any consequences. All participants provided their informed consent to participate in the study by taking the initiative to participate by email, and the study was approved by the Norwegian Centre for Research Data (NSD; Ref. No. 37047).

## Results

The initial reading gave the impression that the participants emphasized how using SIM had made them more emotionally stable and afforded them new experiences with their reactions while working with patients. By way of self-care and managing their personal reactions, the participants also expressed that SIM had helped them to manage countertransference. Moreover, independent of their education and work experience, participants who reported using SIM the most gave the richest interviews. In turn, three themes describing changes in the participants' boundaries, self-care, and emotional reactions toward patients were formulated: managing vulnerability, setting clearer boundaries, and practicing self-care. An overview of the themes, categories, and subcategories, as results from the analysis of all participants' data, appears in Table 2.

**Table 2:** An overview of themes, categories, and subcategories.

**Theme**	**Category**	**Subcategories** **(i.e., relationships between multiple codes)**
Managing personal vulnerability	Dealing with unresolved inner conflicts	Managing affecting childhood experiences Overcoming the need to be better than others or highly successful
	Developing an accepting attitude	Accepting personal emotional reactions Accepting others and situations as they are
	Changing in countertransference reactions	Achieving emotional calmness Managing emotional triggers
Setting clearer boundaries	Being able to set clearer interpersonal boundaries	Feeling non-judgmental and accepting own boundaries Developing new boundary setting skills
	Not tolerating mistreatment	Being assertive in communication Being brave standing up for oneself
	Differentiating oneself from the patient	Being assertive and not confused by patients Being less involved in patients view
Practicing self-care	Reducing stress by striving less	Disengaging from struggle Feeling liberated changing attitudes toward others
	Getting better sleep	Engaging in meditative relaxation

### Managing Personal Vulnerability

The theme of managing vulnerability captures participants' descriptions of resolving unresolved issues and conflicts, including feelings of inadequacy. After beginning to use SIM, participants' ways of dealing with their vulnerability changed to less struggle at work, and more accept of situations, others, and themselves. Along similar lines, participants described acceptance as being part of their new attitude. Changes in experiencing countertransference comprised letting go of a need to be better than others or to be highly successful. For example, Participant 7 described that practicing SIM allowed her, to release the urge to seek perfection for her own success in treatment and interventions:

I can have the intention to reach a goal, but it's not just up to me, then I can only do my best to reach the goal, and either it happens or it doesn't. And then I don't have such strong feelings or expectations about it happening one way or another. I can only do my best.

She also described changes in her countertransference reactions:

It [using SIM] affects me so much... accepting situations as they are, not combating situations so much or becoming so stubborn and … trying to force it through.

In accepting that she was not solely responsible for the treatment goals, she experienced a change in attitude toward meeting with patients. Likewise, Participant 2 described accepting herself in her work after using SIM, which had changed how she felt about herself:

I discovered I wanted to get cred... like I wanted to look good in others' eyes.... I took several rounds on that [SIM]. It made my stress go down, and I stopped messing up so much, trying to be so good. I've become calmer and less stressed.... It's made me a better listener.

She further explained how she had experienced a shift in how she felt about herself and had since felt less insecure as a result:

I've become a lot more secure... very honest, or what to say?... Sincere and honest.... I don't feel the need to be better than I am.... I don't feel the need to transform into anyone other than exactly who I am.

Participant 8, meanwhile, reported being stressed about not having enough time to listen or talk to patients due to a busy schedule performing somatic medical examinations. After trying SIM as a possible solution, she had “worked very concretely” to accept things as they are and to accept her own reactions instead of avoiding the emotions. As a result, she felt less hurt by criticism and disappointment from patients:

It gets different, because you eventually have greater personal development and have more calmness in yourself, both for self-acceptance and in relation to accepting others.

When required to examine patients, she had to accept not having enough time to listen to them:

It boils down to those procedures. I can't do it [the job] in the ideal way, so I've learned to accept that that's just the way it is... I knew that before using SIM, or at least I thought the same thing before, but now I understand it on a deeper level.

After adopting SIM, she had stopped feeling hurt by others or her own criticism as she had before. She also reported accepting that her expectations for managing an ideal way of working were not always realistic. Likewise, as Participant 5 put it, using SIM had made her “so less touchy!”

In another case, Participant 2 described using SIM to manage her reactions when meeting with a patient with grandiosity who was very devaluing toward her:

Then I didn't have any reactions to it [the patient's attitude], although I heard him say very degrading things. I could just listen to what he said, and I didn't contradict him. [Before using SIM] I would have done otherwise. That was what others [colleagues] have done, seen him as their... yeah... “Here comes a difficult guy”... an “enemy.”

Having started to use SIM, she was no longer bothered by the patient's insults. Other participants also experienced changes in their emotional and physical countertransference reactions. Participant 4, for instance, spoke about her emotional reactions and physical fatigue:

I'd be so tired when I left the office that I would fall asleep.... There's a group who drained me a lot, and at the same time, I had so many other feelings as well. I had a need to help them … they never get help. They stay, and I can never help them sort of… It's a very hard group, and it's a group that's triggered me a lot. Then [after practicing SIM]... suddenly I realized that I'm relating to them differently.

She described how, after using SIM, she had experienced emotional and physical changes while working with people with personality disorders and, in the process, had pinpointed the reason for her countertransference:

I think that I grew up with a mother who had a personality disorder and that I have... been too quick to ‘carry people' [take responsibility for other people].... And that comes back to what I learned as a kid... that it's important that I satisfy others.

By engaging in SIM, she had been able to address childhood memories about her relationship with her mother, associate them with her current problems with patients, and, as a result, not be triggered as she had before.

Participant 2 also described how her emotional reactions had changed after beginning to use SIM, particularly how she had reacted less emotionally to patients and others who had previously triggered her anger. She also explained about how she has used rescripting as part of SIM:

It's not like I'm just getting my frustration out.... In a way, I go to the root of it... [I ask] ‘What's it in me that's causing this frustration?'

In turn, she has used imagery rescripting to react differently and gain new insight into the idea of the cause:

And then... I can change it... use my fantasy.... Sometimes it's just like “Let go!”... I don't have such a big reaction.... I experience that I meet all people in another way than I used to.... That's because I manage to keep that calmness in my gut. Then I get a totally different starting point to listen, be calm, and see things a bit from the outside.... It's not so easy to get affected by others or get overwhelmed by other peoples' issues.

### Setting Clearer Boundaries

The theme of setting clearer boundaries captures participants' descriptions of experiences with being able to set clearer interpersonal boundaries than before, not tolerate mistreatment, and differentiate themselves from patients, all due to having used SIM. Participant 4, for instance, discussed changes in how she had set new boundaries for her patients:

For patients with personality disorders, it [using SIM] makes me set boundaries in a completely different way. I don't go along with their nonsense as I used to... and so I don't get myself tangled... and I can help patients to see themselves as well.

She explained that by setting clearer boundaries in her relationships with patients, her involvement with patients became more therapeutic. Using SIM, she had become able to manage emotional triggers that had once made her follow the patient's lead in communication and sacrifice her professionalism. As a result, she expressed feeling less judgmental, more helpful as a professional, and able to define clearer boundaries:

I don't let myself get taken advantage of like I might have been before. I can set boundaries if necessary. It's not okay to be treated badly in any way, even if it's a patient.

Setting clearer interpersonal boundaries thus also meant being brave enough to communicate with patients honestly.

Participant 7 also spoke about how using SIM had helped her be more assertive and set boundaries:

In setting boundaries... all of those emotions and thoughts one might have.... Daring to say what you're thinking and feeling can be hard for different reasons... you might feel ashamed about it [those thoughts and feelings]. But that's what it feels like for me. So, the feeling is real, and, in my case, I actually dare to communicate it.

Having accepted her feelings and experienced increased assertiveness as a result, Participant 7 had become more sincere in her communication.

Participant 3 also described earlier events and how she had needed to change how she was setting boundaries. As a result of engaging in SIM, she reported being able to react but not out of fear or vulnerability in interacting with one patient in particular:

I was so tuned into the patient that I was totally calm. I had worked so much [using SIM] that I didn't put up my guard at all when the patient started yelling.

She additionally described developing new boundary-setting skills instead of acting out *lex talionis*—that is, “an eye for an eye, a tooth for a tooth”—as she once had.

### Practicing Self-Care

Emerging in participants' descriptions of how SIM facilitates wellbeing, the theme of practicing self-care captures how they had reduced stress, accepted their feelings, managed their attitudes, and gotten better sleep. Among participants who described experiences of reduced stress by striving less, Participant 5 reported having gotten rid of her stress and anxiety:

I notice when I'm doing it [SIM].... I do it when I'm stressed out and anxious about something, and I kinda feel like it's not helping just to relax and try to get rid of it... and I immediately feel much calmer afterward. … Yeah, it's not as noisy in my head.

The participants also became calmer, as Participant 1 described:

I've become calmer and more at peace and less annoyed and all of those kinds of feelings. Earlier, it was like I somehow was up here [*gestures and points to her chest and head*]. Now it's more like I'm in my feet.

By comparison, Participant 9 recounted using SIM to be calmer when meeting her patients:

If I'm stressed... then I use it to calm down.... I feel calm... and present.

Participants also reported managing private relationships with SIM and consequently impacting their wellbeing. To them, self-care also means handling their own negative attitudes at work. Participant 7, for instance, felt belittled by her colleagues and described using SIM to manage that situation, which had led her to recognize her negative behavior in her interactions with colleagues and, in turn, alter her attitude, behavior, and relationships with them:

I pulled them down, too, but a lot of the need to pull them down had come from a feeling of being smaller... worse and less meaningful, and so on... than what they were.... When I changed that [by practicing SIM], all of those relationships changed.

She added how her relationships with colleagues were no longer defined by a struggle with feeling less significant and thus needing to belittle them as well. By using SIM, she had discovered that she needed to instead struggle with her sense of inferiority.

As a result of using SIM, most participants described changes in self-care and experiences of wellbeing associated with their ability to manage personal reactions in meetings with patients and others. Participant 10 explained becoming able to deal with anxiety and criticism or correction differently after using SIM:

I used to have problems with anxiety.... I used it [SIM] a lot for that... and it's been really efficient for me.... With bosses and other authoritarian people, I once let it get to me and got very sad if I was criticized or corrected, but now [after practicing SIM] I deal with it differently.

While Participant 10 had experienced a liberating change of attitude toward others' criticism, Participant 6 described how her use of SIM had reduced her stress:

I give myself 25 min [practicing SIM], if I choose to work on a concrete case or just use it for relaxation.... Anyhow, I notice that even if I have all of these things to do, I don't get that feeling of stress.

Last, most participants also described SIM as helping them to get better sleep, including Participant 8:

Once I'd done the 2-day program and used meditation [SIM] every night, I started... like... using it as sleep medication... and now fall asleep in 2 min, my husband says.

## Discussion

In our study, we investigated how healthcare professionals who provide care for patients with mental illnesses have experienced using SIM as an aid for managing countertransference. Overall, our results indicate that participants summarized their experiences along the lines of three themes—managing vulnerability, setting clearer boundaries, and practicing self-care—that interact with one another. For instance, self-acceptance and stress reduction aimed at self-care was described to affect emotional stability in interactions with patients, while practicing self-care (e.g., getting good sleep) could impact stress levels and possibly improve the ability to manage countertransference, a kind of synergy also mentioned by Hayes et al. (24).

### Managing Vulnerability

Managing unresolved unconscious conflicts has been the core of dealing with countertransference since the phenomenon was first addressed (24). In our study, some participants described what might have been the independent management of their unresolved conflicts without supervision or therapy, and those participants were not the ones with the most education or years of experience. For example, Participant 4 described feelings of helplessness because she had learned that it was important to satisfy others and, in her words, “a need to help” people who “never get help.” In using SIM, she had experienced emotional and physical changes while working with patients with personality disorders. As other participants, she described discovering her unresolved inner conflicts and becoming less affected by them after using SIM. Of course, we cannot be sure that SIM was the sole reason for why what seemed to have been unconscious became conscious. Her own reflections in retrospect might have contributed as well.

Instances of accepting oneself, situations, and others had also been experienced due to managing unresolved conflicts. Some of the changes made by participants including overcoming the felt need to succeed or live up to what they now acknowledged was an unrealistic expectation: an underlying need to be successful or a good worker as a hidden agenda obscuring their sincere wish to simply help patients. Indeed, the wish to rescue patients pervades among people who choose to be professional healthcare workers (44, 55), certain types of such wishes may go completely unnoticed as a category of countertransference because they are inherent in such professionals' views of themselves as healers. By using SIM, participants had acknowledged underlying motives, including assessing oneself as being inadequate as a professional healthcare worker, that could unconsciously cause countertransference. Consequently, raising awareness seems to be important for the quality of therapy practice and for therapists' motivation.

Among other results, participants also described what appears to be a change in motivation caused by using SIM. Motivation drives people to make choices to actively pursue the satisfaction of unmet needs, including the need to be accepted by colleagues, to be recognized as skilled and adequate, to avoid the loss of reputation, and to not feel shame (56). Managing such extrinsic motivated needs by accepting oneself as being adequate, as the participants described it, seems to make one's countertransference reactions more formed by patience and accept. Some such changes in acceptance may relate to a positive influence on self-esteem—that is, one's perception of their worthiness and self-respect and the perception that others find them respectable and worthy (57). Our findings suggest that some participants strengthened their self-esteem by using SIM, including the one who became more secure and honest and no longer experienced the need to outperform herself, which she described as a change in herself compared with earlier ways of feeling and being.

Guilt and shame regarding negative reactions to patients are likely to prevent healthcare professionals from dealing with countertransference by creating dissonance in their role and identity of being helping professionals (58). For a person to admit that they have a negative attitude or are judgmental without feeling negatively about it, self-esteem seems to be important. That dynamic suggests that it may be too demanding for some individuals to use SIM for self-disclosure, which may explain why not all participants had engaged in such self-disclosure. To overcome that resistance, a guided process, not merely SIM, may be required. After all, some participants had used SIM only for other specific purposes related to self-care, including getting better sleep. As recommended by Hayes et al. (24), therapy might be assumed to counteract such self-avoidance and yield other results while making unpleasant blind spots visible. That aspect of using SIM merits more research and the development of grounded theory.

### Setting Clearer Boundaries

Managing vulnerabilities seems to be a prerequisite for describing the theme of setting clearer boundaries. In relation to involvement with patients, boundaries in differentiating oneself from others and in showing respect were described. As cited in Gabbard and Lester (59), Lawrence Epstein differentiated boundaries as being thin or thick and permeable or non-permeable. If a healthcare professional has overly thin, permeable interpersonal boundaries, then they may be prone to confusing their internal experiences with the patient's. However, the opposite is the case for professionals with overly thick interpersonal boundaries, who may not experience unconscious communication from patients. Epstein also suggested that individuals with overly thick boundaries may be more prone to seeking solutions in concrete action and less capable of empathy (59). Empathy, an automatically elicited emotional response, is dependent upon the interaction between trait capacities and state influences, and “the resulting emotion is similar to one's perception (directly experienced or imagined) and understanding (cognitive empathy) of the stimulus emotion, with recognition that the source of the emotion is not one's own” p. 7 (60). Thus, being capable of empathy seems to require being able to be in contact with one's emotions, and the cognitive interpretation of the stimulus emotion will add color to the empathetic response. With overly thick boundaries, emotions will not be elicited in the helper and may prompt excessive distance.

As for descriptions of setting clearer boundaries in the findings, whether interpersonal boundaries or boundaries between the self and patients, Participant 4 described the despair of being unable to help a patient. It seems as though her countertransference of feeling sorry for the patient involved her having thin boundaries. Explanations of such helplessness manifested in her interview, and she seemed to have shifted from tolerating mistreatment and being taken advantage to achieve thicker boundaries, thus setting clearer boundaries. She described refusing to follow patients' in to their “nonsense” and not get herself “tangled” in to it. She had become more assertive, considered to be an important factor in relational competency. With such assertiveness, she may now be able to express feelings, opinions, beliefs, and needs openly and clearly, directly, and honestly without adopting an aggressive tone and without feelings that reflect anxiety or violate another's rights (61, 62). A similar dynamic was also experienced by several other participants, some of whom described being mistreated, now acknowledging their thoughts and feelings, and daring to communicate them.

### Practicing Self-Care

Whereas, the other two themes address trait-like attitudes and beliefs, the theme of practicing self-care refers to descriptions of handling disturbances to one's state, handling criticism, reducing stress, and getting better sleep.

Different ways of practicing self-care are essential prerequisites for managing countertransference (24). Acceptance is closely related to the phenomenon of self-compassion (63, 64), which Neff (63) describes as involving three components: accepting one's experiences as a human, being aware of one's experiences but not overwhelmed by them, and understanding instead of judging oneself, especially during difficult times (63). That perspective, as a prerequisite for dealing with countertransference, seems to be present in our findings. For instance, Participant 7, who described belittling and being belittled by her colleagues, had to accept and understand her feelings and behavior to further manage the situation. According to Neff (63) and Neff and Germer (65), an individual has to have compassion for themselves in order to have the emotional resources to have compassion for others. In that light, accepting one's human response appears to be a helpful approach to managing countertransference reactions.

Another relevant feature of the imagery addressed in SIM is how it impacts beliefs about the future. Mental imagery has been shown to increase the likelihood that people believe that something will happen and increase the chance that someone will act on the imagined event. As the event becomes more cognitively available, individuals come to believe more strongly that the event will occur. Individuals fail to recognize, however, that availability is based only on the fact that they were recently induced to access the information (66–68). That dynamic may be of particular concern in relation to wellbeing and countertransference. Having faith and believing more strongly that a beneficial event will happen might lower stress, reduce the tendency to worry, and allow better sleep.

Generating positive images has a powerful, positive impact on emotions (69–72). Overall, such increased emotional stability contributes to managing countertransference (24), which might explain why our participants described being less stressed and having better wellbeing. Participants who had directly managed countertransference toward patients might have also been calmer in knowing that they can manage their emotional reactions.

## Conclusions

We investigated the experiences of healthcare professionals with managing countertransference by using SIM. Our results suggest that the professionals experienced a change in how they managed countertransference via self-care and managing their personal reactions as well. However, there were also differences in the extent and purpose of using SIM among the participants.

### Strengths and Limitations of the Study and Further Research

Our qualitative study afforded an opportunity to capture psychiatric healthcare professionals' experiences with using SIM as an aid for managing countertransference. Strengths of our sampling method included that it netted participants with broad work experience, in different professions, and with varying educational backgrounds. One of our study's limitations, however, was due to convenience sampling, which yielded a sample entirely of women. Because participants had to volunteer to participate in the study, they might be especially interested in the topic. Another limitation was the potential for recall bias about what had happened.

As is the nature of qualitative studies, our research was hypothesis-generating and cannot provide any proof of the benefits of SIM for all healthcare professionals in managing countertransference. Future research should evaluate different models for using SIM in different samples. Beyond that, using validated measures related to countertransference—for instance, the Therapist Response Questionnaire or some form of the Countertransference Questionnaire—would be meaningful to incorporate (73, 74). Last, research should also seek insights into why some individuals succeed more than others in practicing SIM.

## Data Availability Statement

The raw data supporting the conclusions of this article will be made available by the authors, without undue reservation.

## Ethics Statement

The studies involving human participants were reviewed and approved by Norwegian Centre for Research Data (NSD). The patients/participants provided their written informed consent to participate in this study.

## Author Contributions

The study was designed by OA, who also conducted the interviews, while HB acquired approval for the study from the NSD. After OA performed the first phase of analyzing the interviews, all three authors discussed and finished the analysis to optimize the validity of the results. OA wrote the original draft of the manuscript, which was reviewed and revised in collaboration with HB and BN. All authors contributed to the manuscript and approve the submitted version.

## Conflict of Interest

The authors declare that the research was conducted in the absence of any commercial or financial relationships that could be construed as a potential conflict of interest.

## Publisher's Note

All claims expressed in this article are solely those of the authors and do not necessarily represent those of their affiliated organizations, or those of the publisher, the editors and the reviewers. Any product that may be evaluated in this article, or claim that may be made by its manufacturer, is not guaranteed or endorsed by the publisher.
